# Ascites with suspected pneumoperitoneum: a diagnostic conundrum after negative laparotomy

**DOI:** 10.1055/a-2739-2413

**Published:** 2025-11-26

**Authors:** Xinzhu Li, Wanwei Zheng, Zhongguang Luo, Yujen Tseng, Xiao Li

**Affiliations:** 1 159397 Department of Digestive Diseases, Huashan Hospital, Fudan University, Shanghai, China

A 67-year-old man presented with progressive epigastric pain for 1.5 years and ascites for 5 months. Abdominal computed tomography (CT) revealed suspected pneumoperitoneum with large-volume ascites. Given the initial concern for gastrointestinal perforation, an exploratory laparotomy was performed. However, no evidence of perforation was found. Notable intraoperative findings included copious greenish ascitic fluid, diffusely distended small bowel, scattered diverticula, and enlarged mesenteric lymph nodes, which demonstrated reactive hyperplasia upon histopathological examination. Postoperatively, the patient experienced worsening abdominal pain and distension. Follow-up abdominal CT demonstrated persistent massive ascites, with scattered free air ( Fig. 1 ). Positron emission tomography (PET)/CT findings revealed partial small bowel obstruction, without evidence of mass lesions or abnormal FDG uptake ( Fig. 2 ). Laboratory investigations showed normal complete blood count, C-reactive protein, and serum amylase levels, with hypoalbuminea (26 g/L) and microalbuminuria. Analysis of ascitic fluid revealed an albumin level of 17 g/L, with no malignant cells detected. Tuberculosis and other microbial tests were also negative. Ultrasonography showed normal liver morphology with patent portal, hepatic, and splenic venous systems. Cardiac ultrasound, serum proBNP and rheumatological screenings were normal. Fecal calprotectin was markedly elevated at 611.2 µg/g. Colonoscopy was unremarkable. A double-balloon enteroscopy was performed for further investigation ( Video 1 ).

**Fig. 1:**
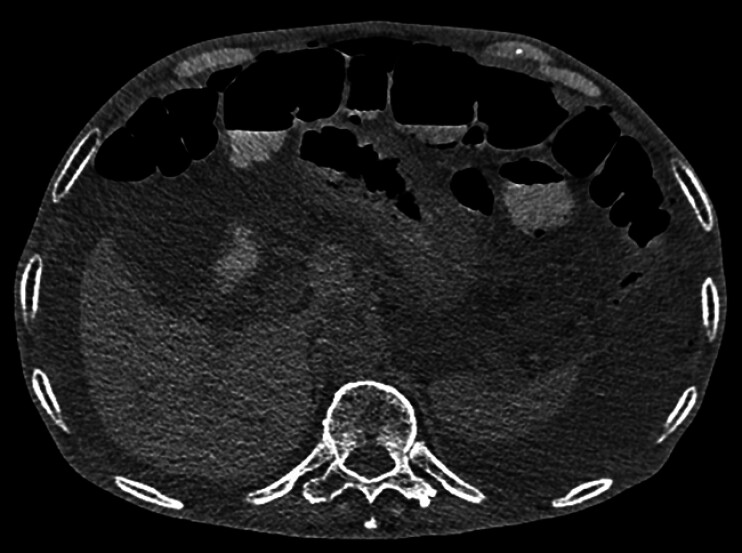
Abdominal CT revealing scattered free gas and significant fluid in the abdominal cavity, as well as partial intestinal dilation with gas-fluid levels.

**Fig. 2:**
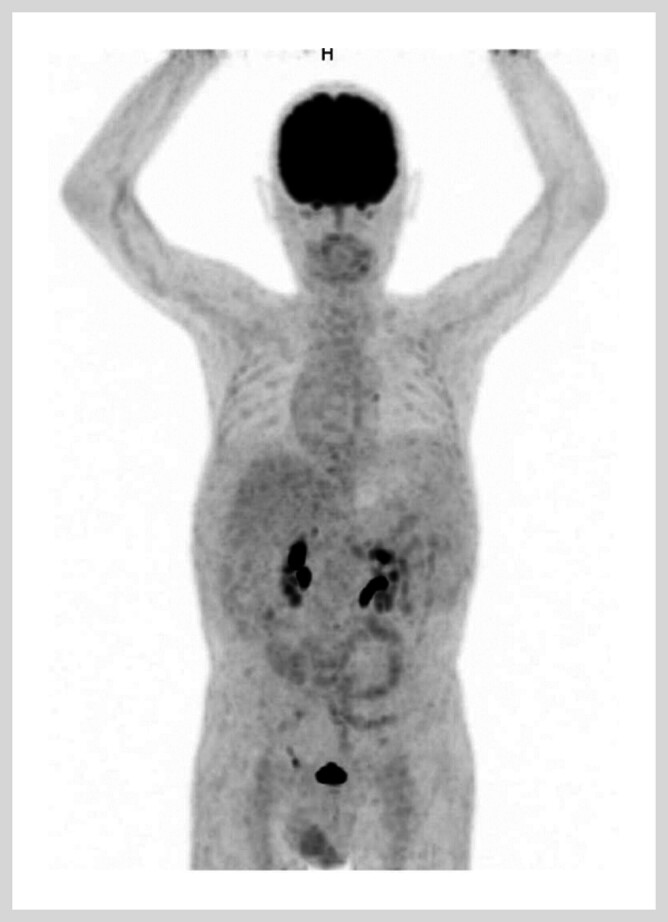
PET/CT revealing incomplete intestinal obstruction with no space-occupying lesions or abnormal FDG increase. Substantial fluid accumulation in the abdominal and pelvic cavities, along with reactive hyperplasia of mesenteric lymph nodes.

Main parts of the double-balloon enteroscopy.Video 1

Small intestinal diverticula are most often detected incidentally during imaging or endoscopic examinations 1 . While typically asymptomatic, small bowel diverticula can cause nonspecific symptoms like early satiety, bloating, and chronic epigastric discomfort, as well as severe complications such as small bowel obstruction, diverticular bleeding, or diverticulitis 2 . However, ascites secondary to jejunal diverticulitis was rarely documented in the literature. Surgical resection of the affected jejunal segment was recommended but the patient declined. A conservative approach with a full-liquid diet with enteral nutritional powder supplementation was initiated. The patient experienced significant improvement in abdominal pain and distension after 3 months, accompanied by significant reduction of ascites upon follow-up ultrasonography.

Endoscopy_UCTN_Code_CCL_1AC_2AF

## Notes

Endoscopy E-Videos https://eref.thieme.de/e-videos
*E-Videos* is an open access online section of the journal *Endoscopy* , reporting on interesting cases and new techniques in gastroenterological endoscopy. All papers include a high-quality video and are published with a Creative Commons CC-BY license. Endoscopy E-Videos qualify for HINARI discounts and waivers and eligibility is automatically checked during the submission process. We grant 100% waivers to articles whose corresponding authors are based in Group A countries and 50% waivers to those who are based in Group B countries as classified by Research4Life (see: https://www.research4life.org/access/eligibility/ ). This section has its own submission website at https://mc.manuscriptcentral.com/e-videos .

## References

[1] Mohanty S Webb SP Uncommon diverticular disease Clin Colon Rectal Surg 2018 31 258 262 10.1055/s-0037-1607970 29942217 PMC6014836

[2] Terada T Diverticulitis of multiple diverticulosis of the terminal ileum Int J Clin Exp Pathol 2013 6 521 523 23411586 PMC3563182

